# Elucidation of Dexmedetomidine‐Induced Analgesic Tolerance Mechanisms in Neuropathic Pain With Modulation of *SGK1* , *NR2A* , and *NR2B* Expression via the Spinal *SGK1* / *NF* ‐ *κB* Signalling Pathway

**DOI:** 10.1111/jcmm.70372

**Published:** 2025-03-18

**Authors:** Wang Huikang, Cao Shiya, Pan Di, Faisal Ayub Kiani, Li Hao, Nan Sha, Lin Xuan, Mahmoud M. Abouelfetouh, Zulfiqar Ahmed, Ding Mingxing, Ding Yi

**Affiliations:** ^1^ College of Veterinary Medicine Huazhong Agricultural University Wuhan China; ^2^ Department of Clinical Sciences, Faculty of Veterinary Sciences Bahauddin Zakariyah University Multan Pakistan; ^3^ Department of Surgery, Anesthesiology, and Radiology, Faculty of Veterinary Medicine Benha University, Moshtohor Benha Egypt; ^4^ Department of Livestock Production, Faculty of Veterinary & Animal Sciences University of Poonch Rawalakot Azad Jammu and Kashmir Pakistan

**Keywords:** analgesic tolerance, mice, *nf*‐*κ b*, *nmdar*, *sgk1*, *α2*‐adrenergic receptor agonists

## Abstract

Neuropathic pain (NP), resulting from nerve damage, is difficult to manage and often requires long‐term treatment. However, prolonged use of pain medications can lead to addiction and reduced effectiveness over time. Understanding drug tolerance is essential for developing improved pain management strategies. Dexmedetomidine (DEX) is effective in targeting the *α2*‐adrenergic receptor, providing relief from pain, especially NP. However, its extended use leads to tolerance and hinders its clinical utility. Herein, we investigated tolerance mechanisms and potential applications of this drug in managing NP. Adult C57BL/6 mice (male) were distributed into DEX Dosage Groups (*n* = 48), DEX Tolerance Model Groups (*n* = 32), *SGK1* Inhibitor GSK650394 Groups (*n* = 48), and *NF*‐*κB* Inhibitor PDTC Groups (*n* = 32) to explore dexmedetomidine's effects on NP and tolerance mechanisms. NP was established via selective ligation of the sciatic nerve branch (SNI), followed by administration of DEX. The results revealed a dose‐dependent analgesic effect of DEX, with significant increases in pain thresholds observed compared to the sham group (*p* < 0.05). Optimal efficacy was found at a dose of 30 μg/kg, indicating its potential as an effective treatment for NP (*p* < 0.05). However, continuous administration of DEX over 13 days induced analgesic tolerance, evidenced by an initial increase in pain thresholds followed by a gradual decrease (*p* < 0.05). Despite an initial efficacy in elevating pain thresholds, the analgesic effect of DEX diminished over time, returning to pre‐dose levels after 5 days (*p* < 0.05). Transcriptome sequencing of spinal cord samples from mice receiving multiple DEX injections revealed differential gene expression patterns, notably upregulation of *SGK1*, *NR2A*, and *NR2B* subunits (*p* < 0.05). Inhibiting *SGK1* mitigated DEX‐induced tolerance, suggesting its involvement in tolerance development (*p* < 0.05). Moreover, *NF*‐*κB* inhibition reversed DEX‐induced tolerance and implicated the *SGK1*‐*NF*‐*κB* pathway in the mediation of analgesic tolerance. To sum up, these findings revealed the molecular mechanism underlying DEX‐induced analgesic tolerance in the NP model and offer potential avenues for future therapeutic interventions.

## 1 Introduction

Disease conditions or physical damage in the somatosensory nervous system often cause neurological ailments and pain. This extremely painful condition affects both humans and animals; hence, it becomes a significant clinical challenge for practitioners [1, 2]. This persistent pain can result from peripheral nerve injuries, medical interventions, or diabetes mellitus. These conditions cause physiological changes in the CNS, leading to hyperalgesia, allodynia, and spontaneous pain. The symptoms contribute significantly to the suffering of affected individuals and impair their quality of life [3].

Central sensitisation plays a crucial role in the onset of NP, involving increased neuronal activity within the CNS [2], which further intensifies this painful sensation. Multiple mechanisms, such as neurotransmitter dysregulation and glial cell activation, are involved in this process, which contribute to the prolonged and often debilitating nature of NP [4]. In addition, elevated peripheral sensitisations also serve as key confounding factors and are characterised by increased responsiveness and reduced threshold of sensory neurones to stimulation [5].

Prevailing clinical‐medical approaches, such as the use of opioids and antidepressants, often exhibit limitations such as side effects and the development of tolerance [6]. Consequently, there is growing interest in alternative therapies like DEX, an *α2* adrenergic receptor agonist known for its pain‐relieving properties with fewer side effects [7]. However, repeated administration of this analgesic drug, such as DEX, often leads to analgesic tolerance, posing a significant challenge in chronic pain management [8].

Despite extensive research, the exact underlying mechanism of the persistence of NP remains elusive; therefore, it hinders the development of effective treatments. Prior studies suggest a potential association between the *SGK1/NF*‐*κB* pathways and the development of opioid tolerance, where their activation induces neuroinflammation and neuronal plasticity [9]. Furthermore, inhibiting *SGK1* or *NF*‐*κB* may attenuate the development of opioid tolerance [10]. However, the genetic basis underlying DEX‐induced tolerance remains unclear in the existing literature.

In our investigation, we probed into the molecular mechanisms underlying DEX‐induced tolerance using an experimental mouse model of NP. Through transcriptome sequencing, we determined significant alterations in gene expression linked to *SGK1*, *NR2A*, and *NR2B* subunits, all pivotal in pain signalling pathway modulation [11]. We focused on how inhibiting SGK1 and NF‐κB pathways influences the development of DEX‐induced analgesic tolerance. Remarkably, by blocking these pathways, we observed a reversal of tolerance, suggesting potential targets for enhancing DEX's therapeutic efficacy in NP management. These findings deepen our comprehension of how DEX influences pain modulation and tolerance development, offering avenues for more effective NP management strategies and improved patient outcomes.

## 2 Materials and Methods

### 2.1 Animals Used, Their Groupings, and Experimental Design

The current investigation used specific pathogen‐free (SPF) male C57BL/6 mice, 8 weeks old, obtained from the Experimental Animal Centre of Huazhong Agricultural University, Wuhan, Hubei Province, China, under the Laboratory Animal Quality adherence (Certificate No. 430727211103208265). Before the initiation of experimental procedures, the housing of experimental mice was maintained under controlled environmental conditions, at a temperature of 24°C ± 2°C with a 12‐h light and dark cycle, and had plentiful access to food and water. One week of acclimatisation was ensured for these experimental mice for optimal health and to minimise stress‐related variables, and their health status was closely monitored to observe any abnormalities.

Following the acclimatisation period, the mice were allocated into distinct experimental groups to investigate the effects of DEX on NP and its associated tolerance mechanisms. The study was structured into four main experimental patterns. Initially, a group of mice (*n* = 40) named the DEX Dosage Group was divided into five subgroups (*n* = 8 each). These subgroups received intraperitoneal injections of DEX at dosages of 0 (sham), 10, 20, 30, and 40 μg/kg. The dosages of DEX and the inhibitors were selected based on previous studies that demonstrated their effectiveness in modulating pain pathways while minimising sedative effects [12]. These dosages are clinically relevant as they optimise therapeutic outcomes for NP management. We ensured that the level of consciousness in the mice was carefully monitored during the experiments, with behavioural assessments confirming effective analgesia without inducing excessive sedation. To confirm this, we observed normal exploratory behaviour and response to external stimuli in the mice, ensuring that the analgesic effects were not confounded by sedation. Following this, the DEX Tolerance Model Group (*n* = 32) was divided into four subgroups (*n* = 8 each). These subgroups received DEX either as a single dose on the last day or as multiple doses administered twice daily over 13 days, with each dose at a concentration of 30 μg/kg (Figure 2A). The group of mice used for our inhibitory studies, such as the *SGK1* Inhibitor Groups (*n* = 48), was further divided into six subgroups (*n* = 8 each) and received an intrathecal injection of the *SGK1* inhibitor GSK650394 at the dose rate of 0.5 nmol/5 μL [13], or a vehicle, 30 min prior to either DEX or saline administration (Figure 5A). Lastly, the *NF*‐*κB* Inhibitor Groups also comprised 48 mice in six subgroups (*n* = 8 each), receiving intrathecal injections of the *NF*‐*κB* inhibitor PDTC at the dose rate of 300 μg/5 μL [14, 15], or a vehicle before the administration of DEX or saline (Figure 6A).

### 2.2 Induction of Neuropathic Pain

The spared nerve injury (SNI) model was established as reported in earlier studies [16, 17], for surgical procedure, mice were anaesthetised with a 1% solution of sodium pentobarbital at a dosage of 50 mg/kg administered via intraperitoneal injection [18]. Following anaesthesia, the surgical area (the right hind limb) was shaved, sterilised, and covered with a surgical drape. The mice were placed in the prone position and secured on the operating table. A 0.5 cm skin incision was made on the outer side of the right thigh. Blunt dissection was performed to expose the sciatic nerve and its three branches: the sural nerve, the common peroneal nerve, and the tibial nerve. The common peroneal nerve and tibial nerve were ligated with 5–0 silk thread at the distal end, and the nerves were transected 2 mm proximal to the ligation site, while the sural nerve was preserved. The muscles and skin were then sutured layer by layer. Sham‐operated mice underwent similar procedures, except for the ligation and transection of nerves. Postoperatively, ampicillin was administered intraperitoneally for 3 days to prevent infection. Mice exhibiting severe self‐mutilation or excessive biting of the affected limb after surgery were excluded from the study.

### 2.3 Behavioural Pain Assessments

Upon establishing the experimental groupings and administering the designated treatments, the behavioural responses of the mice to NP were assessed through a series of standardised tests designed to measure different aspects of pain sensitivity and response. The mechanical paw withdrawal threshold (PWT) was evaluated using an electronic von Frey filament applied to the lateral surface of the right hind paw, which assesses the mechanical pain threshold [19, 20]. Simultaneously, the paw withdrawal latency (PWL) was measured via a hot plate test (IITC, Life Science), providing an indication of thermal pain sensitivity [21, 22]. Additionally, the tail flick latency (TFL), which gauges the response to radiant heat, was determined using a tail‐flick test apparatus [23]. The weight‐bearing ratio (WBR) was assessed using a dual‐inclinometer setup, quantifying changes in the weight‐bearing ability of the mice, which reflects the degree of discomfort or pain experienced [24].

Furthermore, to administer certain drugs directly to the CNS, an intrathecal injection technique was used [25]. This procedure involved restraining the mice and administering injections at the L5‐S1 intervertebral space using a Hamilton micro syringe [25].

### 2.4 Molecular Investigation of Quantitative, Qualitative Expression and Localisation of Genes Involved in Development of DEX Tolerance

Following the behavioural assessments, molecular techniques were employed to further illuminate the cellular and molecular mechanisms underlying NP and the development of tolerance to dexmedetomidine (DEX). The experimental mice were sacrificed using an overdose of sodium pentobarbital (150 mg/kg, intraperitoneal), which is a commonly accepted method for humane euthanasia. Following euthanasia, cervical dislocation was performed to confirm death before tissue collection. Spinal cord tissues were harvested for analysis, beginning with RNA extraction using TRIzol reagent (Invitrogen Corporation, USA). The RNA, which was extracted from spinal cord tissues (L4‐L6 segments of the spinal cord were used for both WB and IF), was subsequently used for quantitative real‐time PCR (qPCR) for the quantitative measurement of the expression of specific genes involved in pathways related to pain and the development of tolerance mechanisms. Primers were optimised to ensure precise targeting of genes of interest. The details of primers, along with their sequence and priming conditions, are detailed in Table 1. The reaction mixture comprised cDNA = 2 μL, REALSYBR Mixture (2×) = 10 μL, both forward and reverse primers = 0.8 μL (10 μmol/μL), and PCR‐grade water = 7.2 μL. The final volume for the qPCR reaction mixture was 20 μL per sample. The following amplification conditions were adjusted for PCR reaction: initial denaturation at 95°C, for 5 min, followed by the 40 cycles of 15 s at 95°C and 60 s at 60°C and 72°C for 20 s. Fluorescence acquisition was subsequently performed after each cycle. Finally, a dissociation curve was generated by increasing the temperature from 65°C to 95°C to verify primer specificity. All samples for each reference gene were run on the same plate to avoid between‐run variations. The relative expression was calculated in accordance with the $2^{-\Delta \Delta C_{t}}$ method [26]. Protein expression in the spinal cord samples was also examined using Western blotting (Table 2). This technique involved homogenising the tissues, followed by separation of the proteins through SDS‐PAGE. This method allows for the quantification and comparison of specific protein levels between different experimental groups, providing insights into the protein dynamics associated with drug administration and NP.

**TABLE 1:** Sequences of primers used for qRT–PCR.

Gene	Accession number	Primer names	Sequences (5'–3')
*Sgk1*	NM_011361	Forward	AGAAAAGGAGCGAGTCCGT
		Reverse	GTGAGGGGTTGGCGTTCATA
*Prkci*	NM_008857	Forward	GCTGTACGAGCTGAACAAGG
		Reverse	GTGCCCCTCTCCGGTAAATG
*Scn2b*	NM_001014761	Forward	CTACATTACCAACCCTCCAGACC
		Reverse	AAGATGACCACAGCCAGGAAA
*Ntsr1*	NM_018766	Forward	GGCAATTCCTCAGAATCCATCC
		Reverse	ATACAGCGGTCACCAGCAC
*Cacna1i*	NM_001044308	Forward	GGCTGGACAGCGTCTCTTTA
		Reverse	GGTGGAAGATGGAGCCAGAC
*Rasa1*	NM_145452	Forward	TTATGATGGGAGGCCGCTATT
		Reverse	CTGCATTGGTACAGGTTCCTT
*NR1*	NM_001372559	Forward	ATCGCCTACAAGCGACACAA
		Reverse	GGATGGTACTGCTGCAGGTT
*NR2A*	NM_008170	Forward	CAAATTCAACCAGAGGGGCG
		Reverse	TGGCAAAGATGTACCCGCTC
*NR2B*	NM_001363750	Forward	CCTACGACACCTTCGTGGAC
		Reverse	TCCATGAATCGGCCCTTGTC
*GAPDH*	AY618199	Forward	CAGAAGACTGTGGATGGCCC
		Reverse	ATCCACGACGGACACATTGG

**TABLE 2:** Drugs and antibodies used in the study.

Drug or antibody name	Company name
Dexmedetomidine	Hangzhou Aladdin Biotechnology Co. Ltd.
PEG300	Medical Chemicals & Engineering (MCE)
PDTC inhibitor	Sigma‐Aldrich Inc. (Saint Louis, MO, USA)
GSK650394 inhibitor	Medical Chemicals & Engineering (MCE)
*NR1* antibody (1:500)	Abclonal Technology Co. Ltd.
*NR2A* antibody (1:500)	Abclonal Technology Co. Ltd.
*NR2B* antibody (1:500)	Abclonal Technology Co. Ltd.
*SGK1* (1:500)	Abclonal Technology Co. Ltd.
*pSGK1*(Bioss, diluted 1:200)	Bioss Biotechnology Co. Ltd.
*p65* Antibody (1:500)	Abclonal Technology Co. Ltd.
*p*‐*p65* Antibody (Abclonal, diluted 1:200)	Abclonal Technology Co. Ltd.
*GAPDH* Antibody (1:1000)	Abclonal Technology Co. Ltd.
Donkey anti‐Rabbit IgG Secondary Antibody	Abclonal Technology Co. Ltd.
Cy3 Goat anti‐Rabbit Secondary Antibody (1:400)	Wuhan Cyvellon Biotech Co. Ltd.
FITC Goat anti‐Mouse Secondary Antibody (1:400)	Wuhan Cyvellon Biotech Co. Ltd.
NeuN Antibody (Proteintech, diluted 1:200)	Proteintech Group Inc.
GFAP Antibody (Proteintech, diluted 1:200)	Proteintech Group Inc.

Additionally, immunofluorescence staining (IF) was performed on sections of the spinal cord to localise and visualise the distribution of targeted proteins within the tissue architecture. This technique confirmed the presence of proteins and provided spatial context to their expression. The reagents used for blotting and IF are listed in Table 2.

### 2.5 RNA Sequencing and Bioinformatics Analyses

To comprehensively understand the transcriptional changes associated with NP and the development of tolerance to DEX, RNA‐sequencing operation was executed at Beijing Biomarker Technologies through the nanopore sequencing technology platform. The Oxford Nanopore technology generated long read sequences and yielded a comprehensive coverage of transcriptomic landscapes. At first, extracted RNA samples from the spinal cord tissues underwent a rigorous quality check for integrity and purity to ensure high‐quality RNA samples for the sequencing process. Following passing of quality checks RNA samples, library construction was initiated. This involved RNA fragmentation into smaller fragments, and then these fragments were undergoing first‐strand cDNA synthesis, where reverse transcriptase was employed to create a complementary DNA (cDNA) strand using the RNA as a template. Second‐strand synthesis was then performed to generate a double‐stranded cDNA, which is necessary for the subsequent sequencing reactions. Each cDNA fragment was then ligated with adapters on both ends to facilitate the binding of the cDNA to the sequencing platform. Sequencing was performed using nanopore technology. This technique involves passing the individual cDNA molecules through a protein nanopore embedded in a synthetic membrane. As the cDNA transits through the nanopore, it causes changes in the electrical conductivity that are detected and recorded. Each change in conductivity correlates to a specific nucleotide sequence, allowing for the real‐time sequencing of the cDNA fragments.

After sequencing, the sequence data was subjected to extensive bioinformatics upstream and downstream analysis to explore differential gene expression (DEGs). This phase involved the alignment of the raw reads to a reference genome of mouse and the determination of gene expression levels and their quantification. Differential expression analysis for genes was then executed to compare the expression profiles of the genes between control and different treatment groups. This identifies genes that were either upregulated or downregulated in response to DEX and its associated treatments. Finally, DEGs were functionally annotated with fold changes in expression, and enrichment analysis was performed to investigate the involvement of these DEGs in biological pathways, providing insights into the molecular mechanisms influenced by the treatments.

### 2.6 Data Analysis

The data collected in this study were analysed using SPSS 26.0 statistical software. Intragroup and intergroup comparisons were performed using two‐way analysis of variance (ANOVA) followed by Bonferroni post hoc test. All the data are presented as (mean ± SD), whereas *p* < 0.05 indicates statistical significance.

## 3 Results

### 3.1 Dexmedetomidine‐Induced Analgesia in Mice With Neuropathic Pain

**FIGURE 1:**
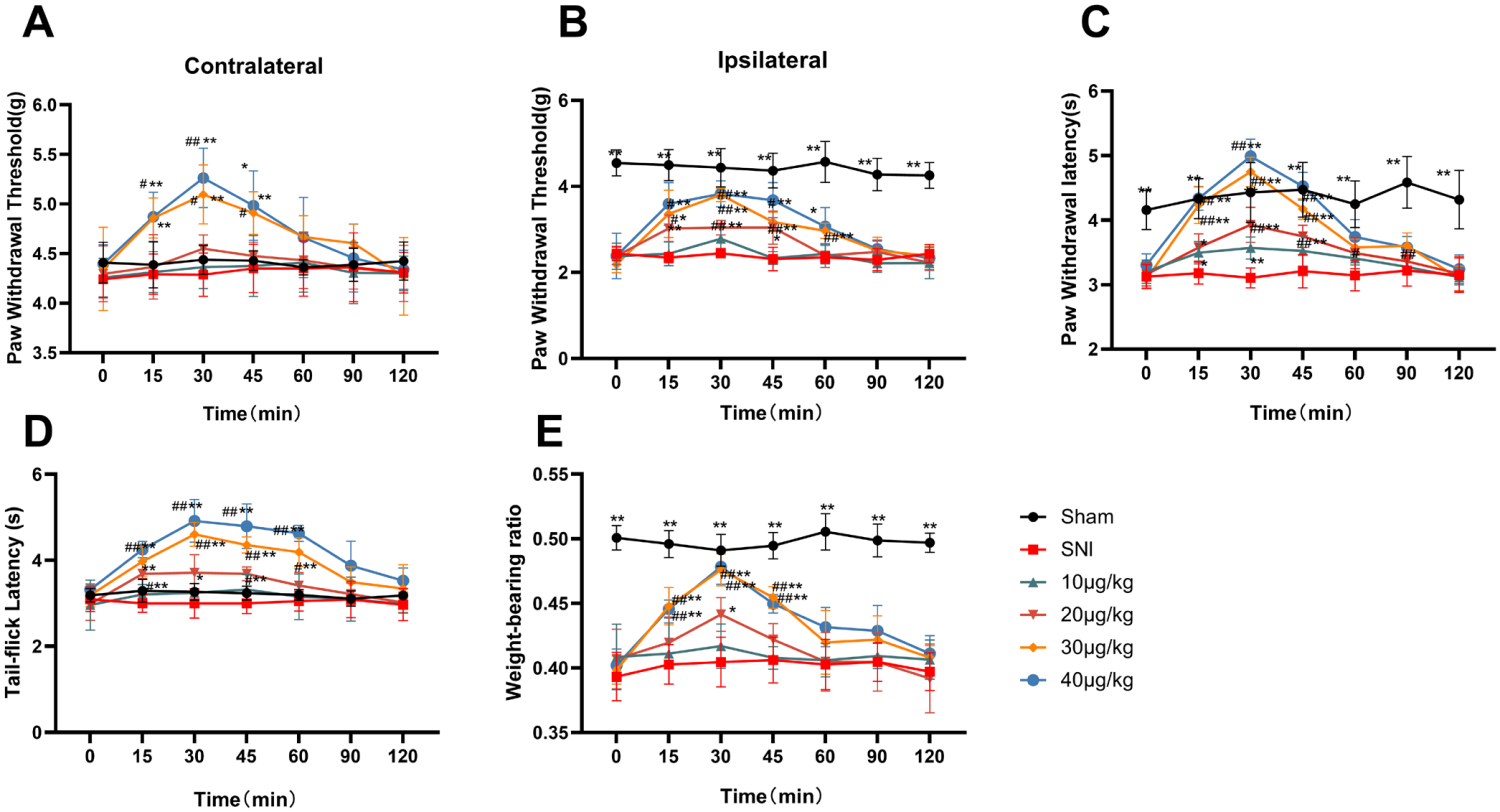
Effect of different doses of dexmedetomidine on pain threshold in SNI model mice (A–E) respectively represented the effects of injection of DEX at different doses on the PWT (contralateral and ipsilateral), the PWL, TFL, and WBR of mice. #*p* < 0.05 indicated that the measured values at each time point after medication had significant differences compared with those before medication, ##*p* < 0.01. *p < 0.05 indicates that the measured value at this measurement time point was significantly different from that of the SNI group, **p < 0.01.

Investigation of the analgesic properties and determination of the optimal dosage of DEX in a mouse model of NP involved the assessment of pain thresholds at different time intervals following the intraperitoneal administration of varying doses of DEX. Compared to the sham group, the paw PWT, PWL, and WBR of the affected limb in the SNI group mice significantly decreased (*p* < 0.01), while the TFL decreased without significant effect (*p* > 0.01). Pain thresholds increased in a dose‐dependent manner within 30 min of administration. Administering 10 μg/kg DEX group showed no significant changes compared to the SNI group, whereas,the 20 μg/kg DEX group demonstrated relative increases in PWT, PWL, TFL, and WBR of the affected limb compared to pre‐treatment. While 30 μg/kg and 40 μg/kg DEX groups exhibited significantly elevated pain thresholds (*p <* 0.01). In addition, a 40 μg/kg dose produced considerable drowsiness along with analgesic effects. These findings confirmed that SNI successfully induced pain sensitivity in mice, and intraperitoneal DEX administration provided significant, dose‐dependent analgesic effects on NP mice without apparent sedative effects at 30 μg/kg. This dose was selected for subsequent experiments (Figure 1A–E).

### 3.2 Continuous Administration of Dexmedetomidine Induces Analgesic Tolerance in Neuropathic Pain Mice

**FIGURE 2:**
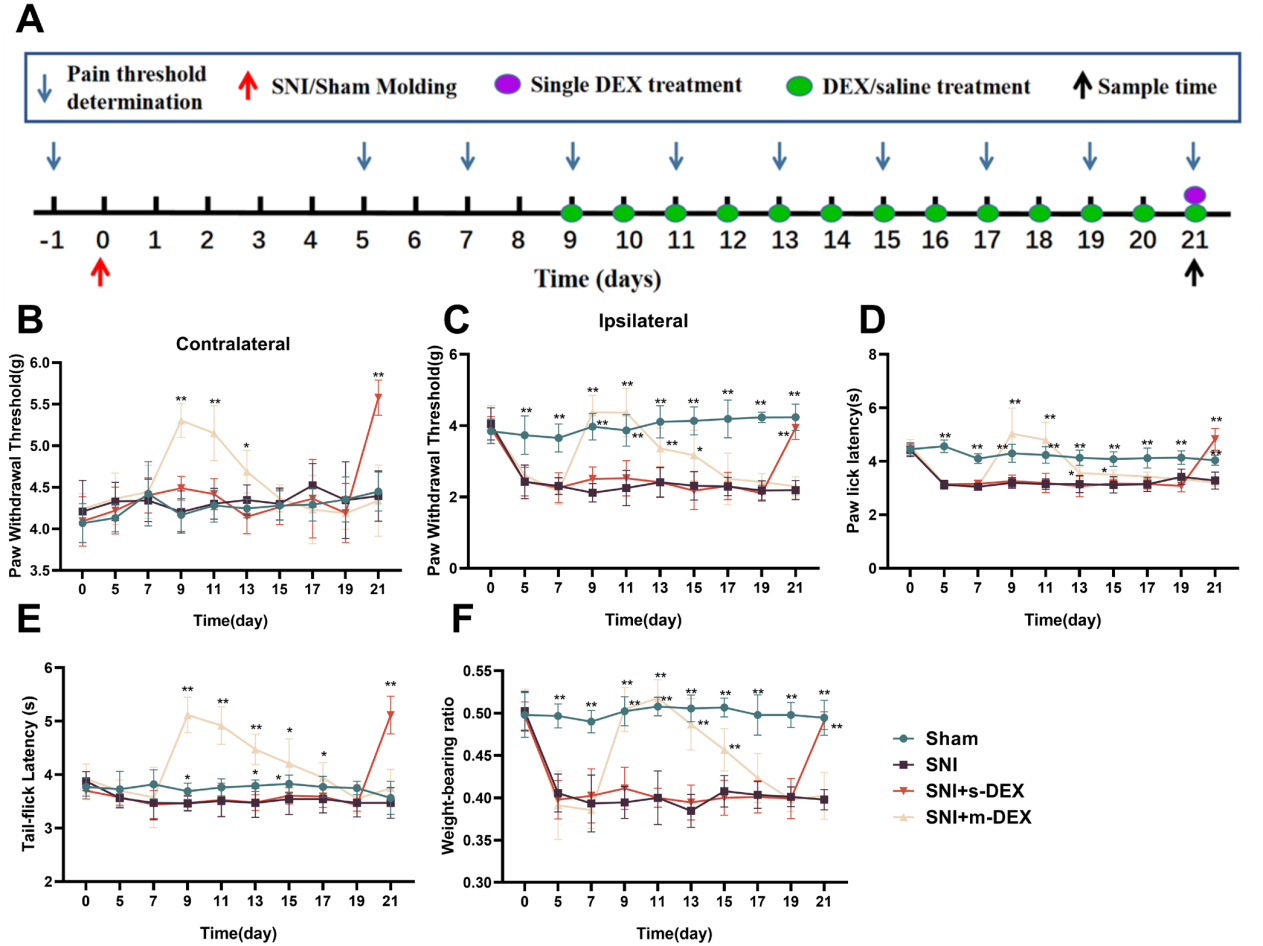
Effect of continuous administration of dexmedetomidine on pain threshold in SNI model mice. (A) Experimental procedure. (B–F) represented the PWT (contralateral and ipsilateral), the PWL, TFL, and WBR of the mice in different treatment groups, respectively. Sham; SNI; SNI + s‐DEX: Single injections of DEX; SNI + m‐DEX: Multiple injection of DEX. *p < 0.05 indicates that the measured value at this measurement time point was significantly different from that of the SNI group, **p < 0.01.

Drug administration for producing an analgesic tolerance model of DEX in mice with NP began on the 9th day following the establishment of the SNI model (Figure 2A). DEX was administrated intraperitoneally (twice a day) for 13 days, and changes in the mice's pain thresholds at different time points were monitored. The results indicate that mice in the SNI + m‐DEX group showed an increase in pain thresholds from Days 1 to 5 after the initiation of DEX administration (*p* < 0.01). However, after 5 days of administration, the pain thresholds gradually decreased, and from Days 11 to 13 of administration, there was no significant difference compared to the SNI group (*p* < 0.01). These findings suggest that short‐term continuous administration of DEX for 1–5 days exhibits good analgesic effects, whereas the analgesic effect significantly diminishes with long‐term continuous administration of DEX for 11–13 days, indicating successful induction of an analgesic tolerance model (Figure 2B–F).

### 3.3 Molecular Investigation of DEX‐Induced Tolerance

#### 3.3.1 Transcriptome Sequencing, Principal Component, Clustering, and Enrichment Analysis for Differential Gene Expression

**FIGURE 3:**
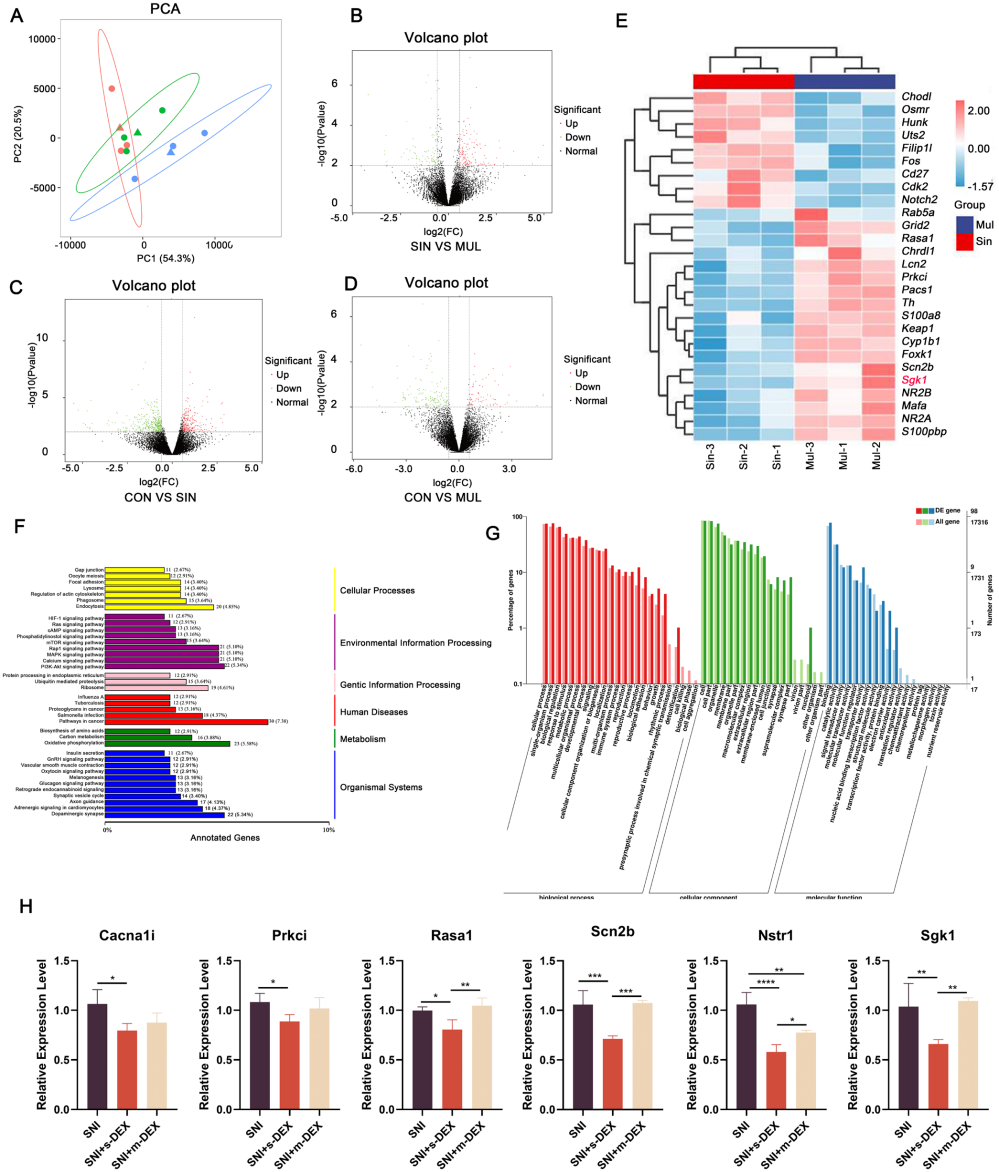
(A) Samples stratification analysis through PCA, (B–D) Volcano Plot Analysis demonstrates the gene expression variances and the significant differences across groups, emphasising the unique molecular signatures induced by different DEX treatment protocols. (E) Clustering Analysis of Differentially Expressed Genes shows significant changes in gene expressions related to pain, neurology, and neuropathic pain pathways in the SIN and MUL groups. (F) KEGG classification diagram of differentially expressed genes of SIN VS MUL (G) Gene ontology annotation classification of Sin vs Mul differentially expressed genes (H) qRT‐PCR validation of DEGs *p < 0.05, **p < 0.01. Sham; SNI; SNI+s‐DEX: single injection of DEX; SNI+m‐DEX group: multiple injection of DEX.

High‐throughput sequencing was employed to further elucidate the mechanisms underlying DEX‐induced analgesic tolerance. Differential gene expression analysis was conducted on the L4‐L6 spinal cord segments of mice across three groups: control (CON), single DEX injection (SIN), and multiple DEX injection (MUL). In addition, principal component analysis revealed significant gene expression differences between the single and multiple injection groups with no overlap, suggesting distinct molecular responses to varying DEX administration regimens. PC1 accounts for 54.3% and PC2 20.5% of the variance of data (Figure 3A). The volcano plot of differentially expressed genes also provided a rapid overview of the differences in gene expression levels between the CON, SIN, and MUL groups, along with their statistical significance (Figure 3B–D). Additionally, detailed screening identified significant variations in gene expression: 614 genes differed between CON and SIN, with 242 upregulated and 372 downregulated; 221 genes differed between SIN and MUL, with 128 upregulated and 93 downregulated; and 222 genes differed between CON and MUL, with 74 upregulated and 148 downregulated (Table 3). The genes, screened in the SIN and MUL groups, include *Grid2*, *Rab5a*, *Lcn2*, *Keap1*, *Cyp1b1*, *Pacs1*, *Sgk1*, *NR2A*, *NR2B*, *Th*, *Foxk1*, *Prkci*, *Rasa1*, *Mafa*, *Scn2b*, *Chrdl1*, *Cdk2*, *Cd27*, *Osmr*, *Filip1l*, *Notch2*, *Hunk*, *Uts2*, *Chodl*, and *Fos* (Figure 3B). Moreover, the clustering analysis of these differentially expressed genes revealed their relation with pain, neurology, neuropathic pain, resistance, and neuronal injury (Figure 3E). Furthermore, the enrichment of these differential genes revealed several significant gene ontology terms and KEGG pathways important for vital aspects of life (Figure 3F,G).

**TABLE 3:** Statistics of the number of differentially expressed genes.

DEG set	DEG number	Upregulated	Downregulated
CON vs. MUL	222	74	148
CON vs. SIN	614	242	372
SIN vs. MUL	221	128	93

*Note:* CON: SNI group; SIN: single DEX injection group; MUL: multiple DEX injection group. Differential gene screening criteria: Fold Change ≥ 1.5 and *p*‐value < 0.01.

### 3.4 Gene Expression Validation Related to SGK1/NF‐κB Pathways

To validate the transcriptome sequencing findings, we considered genes having predetermined roles in pain processing, neurology, neuropathic pain, resistance, and neuronal injury. Six genes, *Rasa1*, *Prkci*, *Cacna1i*, *Nstr1*, *Scn2b*, and S*gk*, were identified as showing significant differences among the control (CON), single DEX injection (SIN), and multiple DEX injection (MUL) groups. We validated these genes using real‐time quantitative PCR to measure their expression levels. The quantitative PCR results (Figure 3H) indicated that the expression of selected genes generally matched the high‐throughput sequencing data, confirming the reliability of the initial findings. Notably, the expression of *SGK1*, which is critical in the development of drug tolerance and NP management [10], decreased after a single DEX injection and increased following multiple injections, underscoring its pivotal role in DEX‐induced analgesic tolerance.

### 3.5 Mechanisms of Analgesic Tolerance Induced by Continuous DEX Administration

**FIGURE 4:**
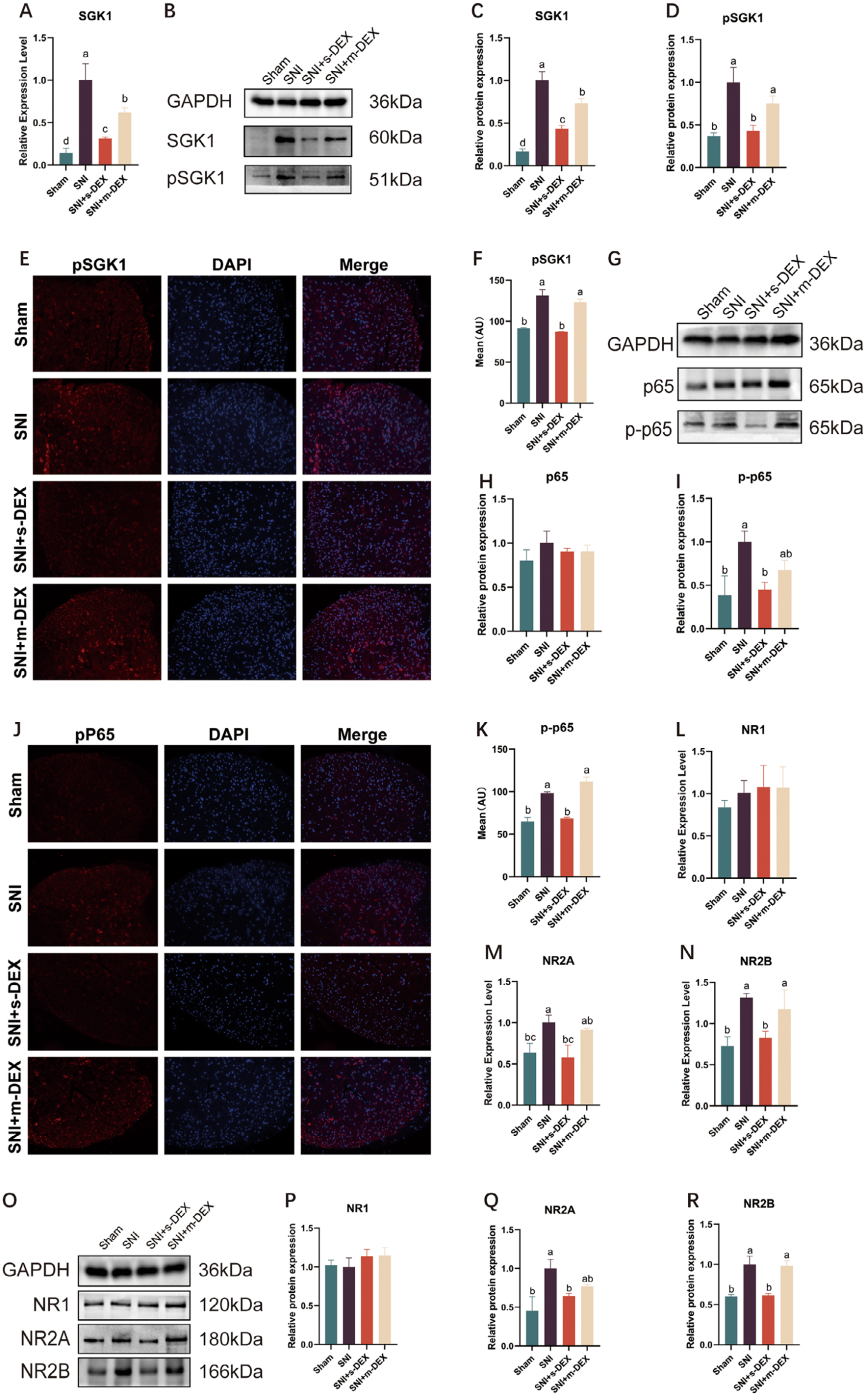
Receptors Expression Profiles in the Spinal Cord of Mice. (A–D) Relative mRNA and protein levels of *SGK1* and *pSGK1* in the spinal cord of mice. (E) Immunofluorescence image of *pSGK1* in the spinal dorsal horn on the modelling side of mice, scale = 200 μm. (F) shows the average fluorescence intensity of *pSGK1* in the spinal dorsal horn of mice. (G–I) Changes in protein levels of *p65* and *p*‐*p65* in the spinal cord of mice. (J) Immunofluorescence image of *p*‐*65* in the spinal cord dorsal horn on the modelling side. Scale bar 200 μm. (K) shows the average fluorescence intensity of *p*‐*p65*. (L–R) Relative mRNA and protein levels of *NR1*, *NR2A*, and *NR2B* in the spinal cord of mice. Data were expressed as mean ± SD. Different letters indicated significant differences among the groups at (*p* < 0.05). *p < 0.05 indicates that the measured value at this measurement time point was significantly different from that of the SNI group, **p < 0.01.

To understand the mechanisms behind analgesic tolerance induced by DEX in mice, we conducted a series of confirmatory experiments. Initially, we examined the interplay between *SGK1* and *NF*‐*κB* pathways and their inhibition. Continuous DEX administration upregulates *SGK1* and *NF*‐*κB* pathways and increases *NMDAR* in the spinal cord of mice [27]. We focused on the NF‐κB pathway due to its critical role in inflammatory signalling and its involvement in the development of analgesic tolerance. NF‐κB interacts with SGK1, regulating molecular targets like NMDA receptor subunits, which influence synaptic plasticity and pain sensitization. Exploring this pathway provides insights into the mechanisms driving DEX‐induced tolerance. To explore this, we examined the expression levels of *SGK1* and phosphorylated *SGK1* (*pSGK1*) at mRNA and protein levels in the mouse spinal cord during DEX‐induced analgesic tolerance progression. Our results showed that *SGK1* levels were elevated in SNI mice compared to sham (*p* < 0.05). However, *SGK1* and *pSGK1* levels were reduced in SNI + s‐DEX (*p* < 0.05). Moreover, after multiple DEX injections, both *SGK1* and *pSGK1* levels increased (*p* < 0.05). Immunofluorescence of *pSGK1* supported the Western blot results (Figure 4A–E).

Meanwhile, continuous DEX administration activates the *NF*‐*κB* pathway. Our experiments confirmed that *SGK1* activation, crucial for NP development, also activates *NF*‐*κB* signalling. We assessed *NF*‐*κB* activity by measuring protein expression levels of *NF*‐*κB* pathway components (*p65* and *p*‐*p65*). There was no significant difference in *p65* expression among groups (Figure 4H). However, *p*‐*p65* expression significantly increased in SNI mice compared to the sham group (*p* < 0.05). A single DEX injection temporarily suppressed *p*‐*p65* expression (*p* < 0.05), but multiple injections caused a rebound in *p*‐*p65* expression, not significantly different from the single injection group (*p* < 0.05). Immunofluorescence staining confirmed these findings, showing a significant increase in *p*‐*p65* fluorescence intensity after multiple DEX injections (*p* < 0.05) (Figure 4G–K). Furthermore, n‐methyl‐d‐aspartate receptor (*NMDAR*) plays a crucial role in pain transmission and synaptic plasticity. Increased *NMDAR* activity can lead to central sensitisation and NP formation. Our study confirmed that NF‐κB activation upregulated NMDAR expression [28]. We measured the mRNA and protein expression levels of *NMDAR* subunits (*NR1*, *NR2A*, and *NR2B*) in the spinal cord of SNI mice. There were no significant changes in *NR1* expression across all groups. Compared to the SNI group, *NR2A* and *NR2B* protein expression significantly decreased after a single DEX administration (*p* < 0.05) but increased after multiple administrations. Significant differences were observed only in *NR2B* mRNA and protein expression levels (*p* < 0.05) (Figure 6L–R).

### 3.6 Inhibition of *SGK1* Pathway Antagonises Development of DEX Analgesic Tolerance

**FIGURE 5:**
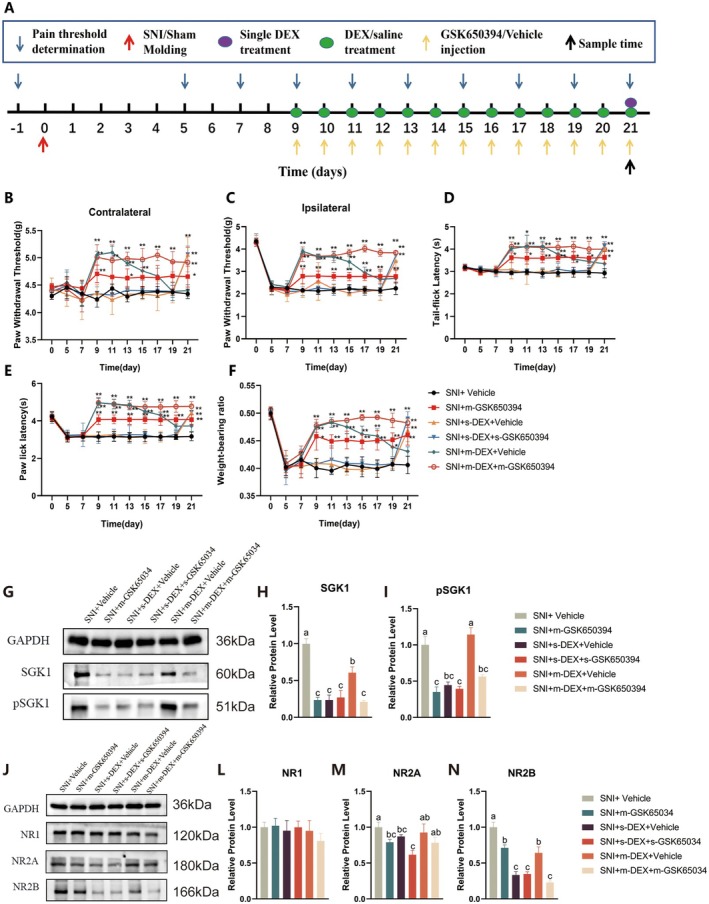
Inhibition of *SGK1* Pathway antagonises development of DEX analgesic tolerance (A) Schematic diagram of the experimental procedure. (B–F) represented the PWT (contralateral and ipsilateral), the PWL, TFL, and WBR of the mice in different treatment groups, respectively. SNI+ Vehicle: SNI group; SNI + m‐GSK650394: Multiple GSK650394 injections; SNI + s‐DEX + vehicle: Single DEX injection; SNI + s‐DEX + s‐GSK650394: Single DEX injection + single GSK650394 injection; SNI + m‐DEX + Vehicle: Multiple DEX injections; SNI + m‐DEX + m‐GSK650394: Multiple DEX + multiple GSK650394 injections. (G) Western blotted bands of *SGK1*, *pSGK1* with *GAPDH* as an internal control. (H and I) relative protein levels of *SGK1* and *pSGK1*. (J) Western blotted bands of *NR1*, *NR2A*, and *NR2B* with *GAPDH* as an internal control. (L–N) relative protein levels of *NR1*, *NR2A* and *NR2B*. Data were expressed as mean ± SD. Different letters indicated significant differences among the groups at (*p* < 0.05). *p < 0.05 indicates that the measured value at this measurement time point was significantly different from that of the SNI group, **p < 0.01.

To further test the role of *SGK1* in DEX‐induced analgesic tolerance in mice, we administered the *SGK1* inhibitor (GSK650394) 30 min before DEX administration intrathecally (Figure 5A). Afterward, pain threshold was assessed in mice. On the 9th day, mice in the SNI + m‐GSK650394 and SNI + m‐DEX + m‐GSK650394 groups showed significantly higher pain thresholds compared to the SNI group (*p* < 0.01), as indicated by increased PWT, PWL, TFL, and WBR (Figure 5B–F). However, by the 11th day, pain thresholds in the SNI + m‐DEX + Vehicle group showed no significant changes. These findings suggest that *SGK1* inhibition effectively reduces pain sensitivity in SNI mice and can counteract long‐term DEX‐induced analgesic tolerance. In addition, for molecular investigation, western blot analysis was conducted after intrathecal injection of the *SGK1* inhibitor to assess *SGK1* expression. The results indicate that mice in both the SNI + m‐GSK650394 and SNI + m‐DEX + m‐GSK650394 groups had significantly lower expression levels of *SGK1* and *pSGK1* in the spinal cord compared to the SNI and SNI + m‐DEX + Vehicle groups (*p* < 0.05). This suggests that long‐term *SGK1* antagonism reduces *SGK1* expression and phosphorylation in the spinal cord of mice tolerant to DEX analgesia. Furthermore, the effect of *SGK1* expression was assessed for spinal *NF*‐*κB* expression in DEX‐induced tolerant mice. To evaluate *NF*‐*κB* expression, western blot analysis was conducted. Results showed a notable decrease in *p*‐*p65* expression in the spinal cord of mice in the SNI + m‐DEX + m‐GSK650394 group compared to those in the SNI + m‐DEX + Vehicle group (*p* < 0.05). This implies that blocking *SGK1* can hinder *NF*‐*κB* pathway activation in DEX analgesic‐tolerant mice. Additionally, the effect of SGK1 inhibition on *NMDAR* expression was also assessed. The outcome of western blotting revealed that the SNI + m‐GSK650394 group exhibited decreased *NR2A* expression in the spinal cord compared to the SNI group (*p* < 0.05). Moreover, both the SNI + m‐GSK650394 and SNI + m‐DEX + m‐GSK650394 groups showed significantly reduced *NR2B* expression compared to the SNI and SNI + m‐DEX + Vehicle groups (*p* < 0.05). These results suggest that blocking *SGK1* inhibits *NMDAR* expression in the spinal cord of SNI mice.

### 3.7 Inhibition of *NF* ‐ *κB* Pathway Antagonises Development of DEX Analgesic Tolerance

**FIGURE 6:**
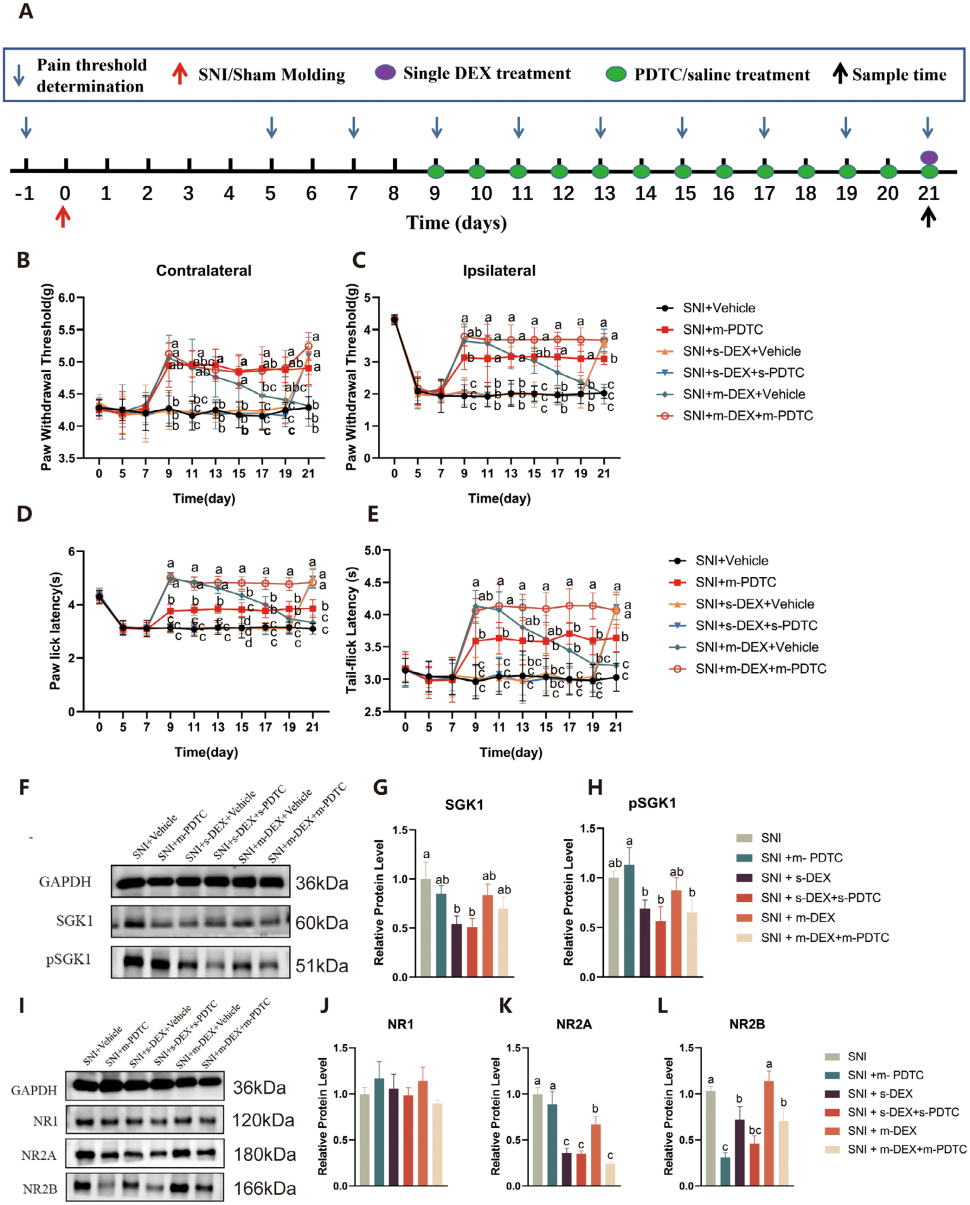
Impacts of PDTC on the expression of *SGK1 pSGK1* in the spinal cord. (A) Schematic diagram of the experimental procedure. (B–E) represented the PWT (contralateral and ipsilateral), PWL, and TFL in different treatment groups, respectively. SNI + Vehicle: SNI group; SNI + m‐PDTC: Multiple PDTC injections; SNI + s‐DEX + vehicle: Single DEX injection; SNI + s‐DEX + s‐PDTC: Single DEX injection + single PDTC injection; SNI + m‐DEX + Vehicle: Multiple DEX injections; SNI + m‐DEX + m‐PDTC: Multiple DEX injections + multiple PDTC. (F) Western blotted bands of *SGK1*, *pSGK1* with *GAPDH* as reference. (G and H) relative protein levels of *SGK1* and *pSGK1*. (I) Western blotted bands of *NR1*, *NR2A*, and *NR2B* with *GAPDH* as internal control. (J–L) relative protein levels of *NR1*, *NR2A*, and *NR2B*. Data were expressed as mean ± SD. Different letters indicated significant differences among the groups at (*p* < 0.05). *p < 0.05 indicates that the measured value at this measurement time point was significantly different from that of the SNI group, **p < 0.01.

Likewise, to further assess the effect of the *NF*‐*κB* pathway we inhibit it with an *NF*‐*κB* inhibitor (PDTC). Continuous intraperitoneal administration of DEX was initiated in SNI mice on the 9th day post‐modelling, and various pain thresholds were assessed at different time points. The pain threshold indicators prior to DEX administration revealed that during Days 1 to 5 of administration, both the SNI + m‐PDTC and SNI + m‐DEX + m‐PDTC groups showed a significant increase in pain thresholds compared to the SNI group (*p* < 0.01), but the thresholds gradually decreased with more DEX administrations on subsequent days. By Days 11 to 13 of administration, there was no significant difference compared to the SNI group, and compared to the SNI + m‐DEX + m‐PDTC group, the pain thresholds were significantly lower (*p* < 0.01). These findings suggest that inhibiting *NF*‐*κB* effectively alleviates pain hypersensitivity and hyperalgesia induced by the SNI model. Moreover, inhibition of the *NF*‐*κB* pathway significantly counteracts the analgesic tolerance induced by long‐term DEX administration in SNI mice. The SGK1‐NF‐κB pathway plays a critical role in modulating *NMDA* receptor subunits, such as NR2A and NR2B, which are essential for synaptic plasticity and pain transmission [29]. Upregulation of this pathway enhances NMDA receptor activity, contributing to central sensitisation and neuronal hyperexcitability in NP. This interaction likely amplifies synaptic transmission, maintaining chronic pain states and tolerance development, making it a promising therapeutic target for preventing DEX‐induced analgesic tolerance. To further check the effects of inhibition of *NF*‐*κB*, western blot analysis of *SGK1*, *pSGK1*, and *NMDAR* was performed after PDTC administration in DEX‐tolerant mice. The results showed that in the SNI and SNI + m‐DEX + Vehicle groups, the expression of *SGK1* and *pSGK1* in the spinal cord of mice in the SNI + m‐PDTC and SNI + m‐DEX + m‐PDTC groups did not significantly change (*p* > 0.05). These results indicate that the NF‐κB pathway is not directly involved in regulating *SGK1* expression. However, antagonising *SGK1* inhibited NF‐κB activation, suggesting *SGK1*'s potential involvement in the formation of DEX analgesic tolerance in SNI mice by activating the *NF*‐*κB* pathway. Additionally, the inhibitory effect of *NF*‐*κB* on *NMDAR* expression was also analysed via western blot. Mice in the SNI + m‐PDTC and SNI + m‐DEX + m‐PDTC groups showed a notable decrease in *NR2B* expression compared to the SNI and SNI + m‐DEX + Vehicle groups (*p* < 0.05). This suggests that inhibiting the *NF*‐*κB* pathway reduces *NMDAR* expression in the spinal cord of SNI mice, counteracting DEX‐induced analgesic tolerance. However, its effects on *NR1* expression remained non‐significant (Figure 6B–L).

## 4 Discussion

Drug tolerance poses a significant challenge in the management of NP, often leading to increased risks of dependence, addiction, and overdose in clinical settings [30]. NP, characterised by its complex aetiology and chronic nature results in drug tolerance due to the lack of targeted treatments for its underlying mechanisms [31, 32]. Alpha‐2 adrenergic receptor agonists, such as DEX, are favoured for their safety profile, reversibility, and relatively fewer adverse effects compared to other analgesics [33]. Despite these benefits, the prolonged use of DEX is associated with diminished analgesic efficacy and the onset of tolerance, the exact genetic and molecular mechanisms of which remain insufficiently understood and have not been validated in model animals.

In this study, we utilised the SNI mice model to replicate the clinical presentation of chronic NP and assess DEX‐induced analgesic tolerance [16]. The SNI model is well‐established for inducing stable nociceptive hypersensitivity, evidenced by consistent pain threshold measurements across the experimental timeline [34]. These effects were measured through the use of indicators such as PWT, PWL, TFL, and WBR to evaluate the analgesic effect of DEX and nociceptive hypersensitivity in the SNI model, and WBR was used to evaluate the SNI modelling and the change of right and left hind limb weight‐bearing after drug administration. This model facilitated a reliable platform for investigating the pharmacodynamics of DEX and the mechanisms underpinning its tolerance [35].

Our findings regarding DEX administration corroborate with the earlier findings, which found that DEX achieved analgesic effects in a dose‐dependent manner. Our findings demonstrate that intraperitoneal administration of 30 μg/kg DEX achieved significant analgesia without excessive sedation, which is quite similar to the findings in another study that found that 30 μg/kg DEX was sufficient to cause analgesia without producing oversedation [36]. This dosage was selected for subsequent experiments based on its optimal balance of efficacy and minimal sedation, aligning with previous research indicating a dose‐dependent analgesic effect of DEX [37, 38]. Our data revealed that while DEX provides substantial analgesia initially, its prolonged use leads to significant tolerance, evidenced by a marked decline in pain thresholds over a 13‐day administration period, suggesting that prolonged use of DEX in the mouse SNI model leads to analgesic tolerance.

To clarify the molecular mechanisms driving DEX‐induced analgesic tolerance, we performed high‐throughput sequencing and differential gene expression analysis on spinal cord samples from SNI mice. This analysis identified significant enrichment of genes within the *PI3K*‐*Akt* and *MAPK* signalling pathways, implicating these pathways in the development of DEX tolerance. Notably, *SGK1* emerged as a key player, with its expression significantly upregulated in DEX‐tolerant mice [39]. This finding aligns with previous studies suggesting that *SGK1* is involved in neuronal plasticity and central sensitisation processes [40, 41, 42].

The role of *SGK1* in mediating DEX tolerance was further supported by our experiments utilising the *SGK1* inhibitor GSK650394. Administration of GSK650394 effectively mitigated DEX tolerance, reducing both mRNA and protein levels of *SGK1* and its phosphorylated form (*pSGK1*) in the spinal cord. This result underscores the pivotal role of *SGK1* in the development of DEX tolerance, echoing findings from studies on morphine tolerance where prolonged treatment induced *SGK1* phosphorylation without altering its expression levels [10] Our study highlights the unique role of SGK1 in DEX‐induced tolerance compared to morphine.

Our findings revealed the critical role of the *NF*‐*κB* pathway in mediating DEX‐induced analgesic tolerance, with *SGK1* emerging as a central regulatory factor. *NF*‐*κB* activation was evidenced by increased levels of phosphorylated *p65* (*p*‐*p65*) in the spinal cord of mice after multiple DEX injections, suggesting that prolonged DEX administration triggers *NF*‐*κB* signalling. This finding aligns with previous studies demonstrating that *NF*‐*κB* plays a central role in chronic pain and inflammation by regulating the expression of various genes involved in these processes [29, 43, 44]. The inhibition of *SGK1* using GSK650394 significantly reduced the levels of *p*‐*p65*, indicating that *SGK1* is upstream of *NF*‐*κB* in this pathway and mediates its activation. This inhibition also led to a decrease in *NMDAR* subunits *NR2A* and *NR2B*, which are implicated in pain transmission and central sensitisation, thereby reducing DEX‐induced tolerance [10, 40]. Furthermore, inhibiting *NF*‐*κB* with PDTC attenuated the upregulation of *NR2B*, further confirming the pathway's involvement in analgesic tolerance. These findings suggest that targeting the *SGK1/NF*‐*κB* axis could be a promising strategy for mitigating tolerance to analgesics like DEX and improving long‐term pain management [45, 46].

Several recent studies have also explored the crucial role of *SGK1* and *NMDA* receptor subunits *NR2A* and *NR2B* in neuropathic pain. They provided *SGK1* evidence that spinal is involved in the development of neuropathic pain caused by nerve injury. Blocking the activation of *SGK1* pharmacologically was found to ameliorate allodynia in a spinal nerve ligation (SNL) rat model [9]. Additionally, *SGK1*‐dependent *HDAC4* phosphorylation and cytoplasmic retention, which may alter nociception‐associated gene expression, were shown to contribute to SNL‐induced allodynia. The *NMDA* receptor subunits *NR2A* and *NR2B* have also been implicated in neuropathic pain, with *NR2B* expression in the dorsal root ganglia (DRG) differentially regulated following peripheral nerve injuries [47].

Likewise, the high‐throughput sequencing data of this study further revealed notable upregulation of *SGK1*, *NR2A*, and *NR2B*, which are crucial in the development of DEX‐induced analgesic tolerance. *SGK1*, being a key player in neuroinflammatory processes and neuronal plasticity [10], likely contributes to tolerance by enhancing these pathways. The increased expression of *NR2A* and *NR2B*, subunits of *NMDARs* in our study, suggests their involvement in sustaining central sensitisation and higher pain sensitivity during prolonged DEX administration. The *SGK1/NF*‐*κB* pathway may interact with *NMDAR* mechanisms to sustain analgesic tolerance, suggesting new therapeutic targets.

Taken together, our study explains the critical role of spinal *SGK1* in DEX‐induced analgesic tolerance through the activation of *NF*‐*κB* signalling and subsequent modulation of *NMDAR* expression. Targeting *SGK1* offers a promising avenue for mitigating drug tolerance, highlighting the need for further research to develop therapeutic interventions aimed at improving long‐term analgesic efficacy.

However, the current study has some limitations that can be addressed in future experiments. As the use of animal models, specifically male C57BL/6 mice, may not fully replicate the complexity of NP and drug tolerance in humans, particularly due to physiological and genetic differences between species. In addition, the use of male mice also limits the generalisability of the findings across sexes, as females may exhibit different responses to DEX and its associated pathways. Moreover, the short duration of the study does not capture the long‐term effects of DEX, which may be critical in chronic pain management. Future research should involve longer study durations, inclusion of female subjects, and exploration of how these findings translate to human physiology to enhance the clinical relevance of the results. These include the genetic manipulations or gene knockouts that could provide deeper insights about the phenomenon of tolerance development. Moreover, we did not examine how DEX interacts with other pain medications, which could be important for clinical use. Investigating these interactions in future research could improve pain management strategies. Finally, while we focused on the *SGK1/NF*‐*κB* pathway, other molecular pathways and factors may also play a role in DEX tolerance. Future research should explore these areas to develop better therapies for managing drug tolerance and enhancing pain treatment.

## 5 Conclusion

This study successfully explored mechanisms underlying DEX‐induced analgesic tolerance in an NP model using male C57BL/6 mice. Our findings reveal that continuous administration of DEX leads to significant analgesic tolerance, mediated through the upregulation of *SGK1* and activation of the *NF*‐*κB* signalling pathway. Transcriptome analysis identified differential gene expression, with notable increases in the expression of *SGK1* and *NMDA* receptor subunits *NR2A* and *NR2B*. Inhibition of *SGK1* with GSK650394 mitigated *NF*‐*κB* activation and reduced *NMDA* receptor expression, effectively attenuating DEX‐induced tolerance. Similarly, inhibiting *NF*‐*κB* with PDTC also diminished *NR2B* upregulation, further confirming the critical role of the *SGK1/NF*‐*κB* pathway in this process. Targeting the *SGK1/NF*‐*κB* pathway could help to prevent or reverse analgesic tolerance, improving the long‐term efficacy of DEX in chronic pain management.

## Author Contributions

**Cao Shiya:** conceptualization (equal), data curation (equal), methodology (equal), software (equal), writing – original draft (equal). **Wang Huikang:** investigation (equal), methodology (equal), writing – original draft (equal). **Faisal Ayub Kiani:** formal analysis (equal), methodology (equal), writing – original draft (equal). **Pan Di:** conceptualization (equal), formal analysis (equal), investigation (equal), methodology (equal), writing – original draft (equal). **Li Hao:** formal analysis (supporting), methodology (supporting). **Nan Sha:** formal analysis (supporting), methodology (supporting). **Lin Xuan:** formal analysis (supporting), investigation (supporting), methodology (supporting), software (equal). **Mahmoud M. Abouelfetouh:** conceptualization (supporting), formal analysis (supporting), investigation (supporting), methodology (supporting), software (supporting). **Zulfiqar Ahmed:** data curation (supporting), methodology (supporting). **Ding Mingxing:** conceptualization (lead), funding acquisition (lead), project administration (lead), writing – review and editing (lead). **Ding Yi:** conceptualization (lead), investigation (lead), project administration (lead), resources (lead), supervision (lead), validation (lead), writing – review and editing (lead).

## Ethics Statement

The study was conducted in accordance with the ethics committee of Huazhong Agricultural University (430727211103208265).

## Conflicts of Interest

The authors declare no conflicts of interest.

## Data Availability

The raw data in this study were deposited in the NCBI database under accession number PRJNA1113302.
